# Large scale wheat data integration improves genomic prediction accuracy with the potential to facilitate international breeding collaborations

**DOI:** 10.1038/s42003-026-10150-x

**Published:** 2026-04-28

**Authors:** Abdulqader Jighly, Reem Joukhadar, Gabriel Keeble-Gagnere, Irene van den Berg, David Chisanga, Deepmala Sehgal, Susanne Dreisigacker, Matthew Hayden, Hans Daetwyler, Jennie Pryce, Sukhwinder Singh, Richard Trethowan, Mike Goddard

**Affiliations:** 1 Agriculture Victoria, AgriBio, Centre for AgriBiosciences, Bundoora, VIC Australia; 2 International Maize and Wheat Improvement Center (CIMMYT), Texcoco, Mexico; 3 School of Applied Systems Biology, La Trobe University, Bundoora, VIC Australia; 4 United States Department of Agriculture, Agricultural Research Service, Subtropical Horticulture Research Station, Miami, FL USA; 5 School of Life and Environmental Sciences, Plant Breeding Institute, Sydney Institute of Agriculture, The University of Sydney, Narrabri, NSW Australia; 6 Faculty of Veterinary and Agricultural Science, University of Melbourne, Parkville, VIC Australia; 7 Present Address: AgriSapiens PTY LTD, Doncaster, VIC Australia

**Keywords:** Agricultural genetics, Plant breeding

## Abstract

Effective data integration across diverse sources is essential for maximising genetic gain and accurately predicting plant performance in modern agriculture. However, individual breeding programs often face significant logistical and financial barriers when attempting to test large, diverse populations across a wide range of global environments. Here we show that synchronising genomic data from two prominent wheat breeding programs with a total of 11,609 wheat accessions that were evaluated across 79 environments establishes a robust platform that significantly improves genetic prediction accuracy and the power to identify complex trait associations. By developing a computationally efficient statistical model, we demonstrate that combining these massive datasets increases prediction accuracy by up to 13% while drastically reducing the time and computational resources required for analysis. This collaborative approach leverages existing investments and taps into broader genetic diversity to overcome the geographical and resource limitations of isolated programs. Our findings highlight the transformative power of collective data integration and provide a practical framework to revolutionise current breeding strategies. Ultimately, our framework has the potential to facilitate international cooperation and streamline the development of climate-resilient varieties, thereby facilitating more sustainable agricultural practices and strengthening global food security.

## Abstract (web summary)

Collaborative data integration and efficient 3GS modeling across global wheat programs enhance genomic prediction accuracy and statistical power, providing a framework for rapid development of climate-resilient varieties for food security.

## Introduction

Recent advances in crop genomics platforms have facilitated the genotyping of large breeding populations at a reasonable cost^1^. Consequently, breeding programs are increasingly using genomic prediction for the design of future crosses and the selection of candidates based on their genetic merit for continued product development^2^. The success of this approach is governed by the accuracy of prediction for different traits across a wide range of environmental conditions. Prediction accuracy is largely affected by the size of the reference population, the heritability of the trait, the genotyping density, and the relatedness between the reference population and the selection candidates^3,4^. Therefore, it is critical that the reference population is large and diverse^5^. This can improve the selection efficiency, especially for traits with low heritability, and increase the probability that the genetic diversity of selection candidates is represented in the reference population^6^. It is also important to evaluate materials across a range of environments to better predict the germplasm performance and accelerate the selection of climate resilience cultivars^7^.

Achieving a reliable and ideal reference population size has logistical and financial constraints. It might not be affordable for a single breeding program to test tens of thousands of individuals across variable environments. Moreover, local breeding programs might be restricted to specific geographical zones with limited ability to evaluate germplasm outside these zones^8^. Variant effects calculated through genomic prediction in one breeding program can be used to predict the performance of less related lines developed in another breeding program. However, to attain more accurate inter-breeding program prediction, analyzing the data from multiple breeding programs together with proper prediction models that consider genotype by environment interaction (GxE) is required^9^. Such models were previously shown to improve prediction accuracy, especially when used across different populations, since they utilize wider genetic diversity to train the prediction equation as well as increase the population size in the integrated analysis. This facilitates more precise adoption of external germplasm across multiple breeding programs^10,11^.

Integrating data across breeding programs may not always be straightforward unless all programs were genotyped with the same platform or sequenced at the whole genome level^12^. Breeding programs differ in their genotyping platforms and sometimes have very limited common genotyped loci. However, provided genomic data from different breeding programs can be imputed to a common higher-density platform, then a common set of high-quality imputed variants across all breeding programs can be used for subsequent analyses^13^. Another obstacle with data integration is the computational complexity associated with the increased size of the reference data, especially when increasing the number of environments. For standard GxE prediction models, the computational time usually increases quadratically as the number of environments expands, which limits their applications for big data.

Here, we integrated data from two large pre-breeding programs, (1) the Australian wheat heat tolerance per-breeding program at the University of Sydney, which was genotyped with 90K^14^ or 40 K SNP chips^15^, and (2) the International Maize and Wheat Improvement Center (CIMMYT) pre-breeding program, which was genotyped with a genotyping by Sequencing (GBS) approach^16,17^. We also compared standard genomic prediction models to an improved version of the computationally efficient model 3GS^18^, which can be applied to unbalanced data and has linear computational complexity as the number of reference environments increases.

## Results

### Genotype imputation

The primary aim of our study was to facilitate collaboration among breeding programs by enabling the seamless integration of their data. We demonstrated this by using genotyping and phenotyping data from two well-established wheat breeding programs: one belonging to the University of Sydney (USyd) and the other belonging to the International Maize and Wheat Improvement Centre (CIMMYT). The first step was to synchronize the genotypic data generated with different genotyping platforms through a genotype imputation process. Specifically, we imputed a high-density exome capture SNP set^19^ for each of the genotyping platforms used by the USyd and CIMMYT breeding programs. Imputation accuracy results are presented in Table 1. The three genotyping platforms (90 K, 40 K, and GBS) showed generally high accuracy of 0.96, 0.92 and 0.75, respectively.

**Table 1:** Imputation accuracy for the three integrated germplasms

Germplasm	*N*	Accuracy
USyd-90 K	3082	0.96
USyd-40 K	3005	0.92
CIMMYT-GBS	6258	0.75

The imputation process across the three platforms (Table S1) resulted in an average overall accuracy ranging from above 0.9 for low-MAF variants (MAF < 0.1; a total of 8383 SNPs) to 0.73 for high-MAF variants (MAF > 0.4; a total of 7068 SNPs). Considering each genotyping platform, the 90 K and 40 K platforms maintained high accuracies across all MAF ranges. However, for the GBS platform, concordance accuracy was notably lower for variants with higher minor allele frequencies, reaching 0.56 for SNPs with a MAF > 0.40 (Table S1). This indicates that for common variants in the GBS dataset, imputation performance was slightly better than random assignment. Despite this, a common set of 32,822 high-quality imputed SNPs was successfully identified across all platforms for subsequent analyses.

**Fig. 1:**
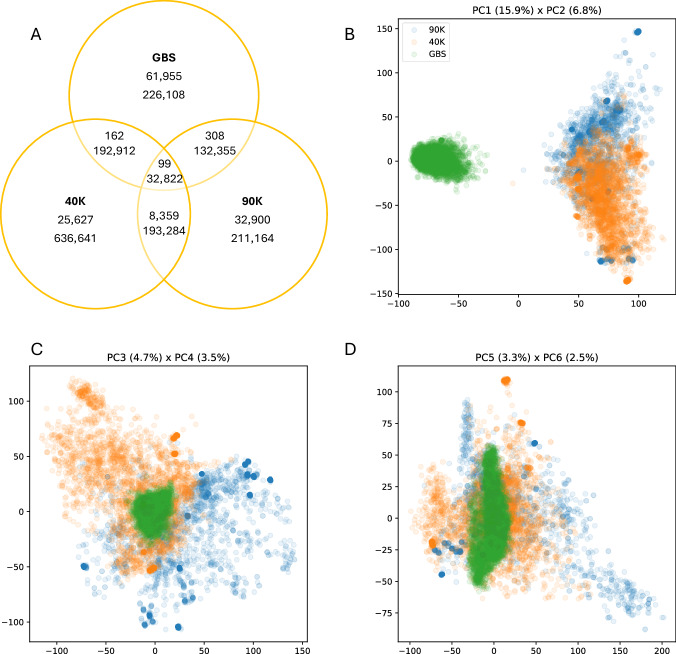
SNP and PCA analysis across the three genotyping platforms (90 K, 41,666 SNPs, 40 K, 26,196 SNPs, and GBS, 70,575 SNPs). **A** Venn diagram showing the common number of SNPs across the three platforms. The upper number represents the number of SNPs before imputation, and the lower number represents the number of SNPs after imputation. **B**–**D** PCA analysis (PC1 to PC6) using the common set of SNPs across the three genotyping platforms.

Subsequently, we identified and selected common SNPs across these three distinct datasets, which resulted in 32,822 common SNPs (Fig. 1a) that were used for subsequent analyses. We used principal component analysis (PCA) to evaluate the consistency in clustering patterns of each genotyping platform before and after the imputation, and to visualize the relationships among individuals within and between the breeding programs. This could be because the reliance is only on the shared imputed variants across all platforms. Across genotyping platforms, the first PC distinctly separated individuals from the two breeding programs (Fig. 1b). PC2, PC3, and PC4 demonstrated partial separation, particularly among individuals genotyped using the 40 K and 90 K SNP chips (Fig. 1b, c). Subsequent PCs (from 5 to 6) revealed overlapping patterns across the three datasets (Fig. 1d). Linkage disequilibrium decay for USyd (genotyped with 40 K and 90 K) and CIMMYT (genotyped with GBS) programs, as well as the combined data, can be found in Fig. S1. The USyd program decayed below the critical threshold at 16–17 Mb, while the CIMMYT program decayed at 12–13 Mb. Interestingly, when analyzing both programs together, the LD decayed below the critical threshold at around 10 Mb.

### Genomic prediction

We conducted a comprehensive comparison of prediction models for ten important traits, using both the individual breeding programs as a reference population (Sub) and a combined across-program reference population (All). This included Genomic Best Linear Unbiased Prediction (GBLUP) models that accounted for distinct environments as fixed effects (GE), or considered genotype-environment interactions (GxE). It also included the newly developed 3GS model, which also considers GxE and is much faster than the standard GBLUP model. When analyzing each breeding program independently, the GxE model was observed to consistently outperform the GE model, with an average prediction accuracy of 0.47 compared to the average prediction accuracy of the GE model of 0.39 (Table 2). When combining data from both breeding programs, the average prediction accuracy of the GE model for yield improved to 0.41; however, the 3GS model notably improved to 0.50. Interestingly, for the three traits involving over 50 different environments (heading date, plant height, and grain yield), the computation of the GxE model failed to finish even after allocating extensive computational resources (500 GB of RAM and over a day of processing time). This reflects the computational challenge for standard GBLUP models when applied to large datasets. In contrast, the entire analysis with the 3GS model for grain yield across 79 environments was completed in less than one hour with 10 GB of RAM, underscoring the computational efficiency of the 3GS model in handling large datasets.

**Table 2:** Average genomic prediction accuracy across different environments for ten commercial traits using three different models: GE, GxE, and 3GS

Trait	No records	NoEnv Usyd	NoEnv CIMMYT	GE		GxE		3GS	
				Sub	All	Sub	All	Sub	All
HD	53,158	25	38	0.29	0.35	0.39	NA	0.41	0.46
MD	31,567	20	14	0.44	0.48	0.47	0.53	0.48	0.54
PH	41,404	21	35	0.39	0.39	0.44	NA	0.44	0.47
Prot	14,586	20	1	0.45	0.44	0.54	0.54	0.53	0.55
Screen	9696	22	0	0.32	NA	0.45	NA	0.47	NA
TKW	16,314	24	1	0.60	0.62	0.64	0.65	0.65	0.65
YLD	58,427	30	49	0.21	0.25	0.37	NA	0.37	0.41
Lr	4527	5	0	0.45	NA	0.48	NA	0.48	NA
Sr	8074	5	1	0.35	0.38	0.43	0.46	0.44	0.47
Yr	11,498	5	2	0.41	0.44	0.47	0.50	0.47	0.50

*NoEnv* number of environments, *Sub* average prediction accuracy results for each breeding program independently, *All* average prediction accuracy results when analyzing both breeding programs, *NA* analysis was not possible due to the lack of records in one dataset or due to computational limitation, *HD* heading date, *MD* maturity date, *PH* plant height, *Prot* protein content, *Screen* screening percentage, *TKW* thousand kernel weight, *YLD* grain yield, *Lr* leaf rust, *Sr* stem rust, *Yr* yellow rust.

Given that the PCA analysis revealed distinct clustering patterns for the different genotyping platforms, we assessed the impact of imputation on prediction accuracy by repeating the prediction accuracy analysis while incorporating the genotyping platform as a fixed effect covariate. This approach aimed to ensure the imputation process had no discernible effect on prediction accuracy and that the observed clustering results were due to true differences among the populations. Therefore, the clustering is mainly a result of the differences in structure and allele frequency among the populations. This assessment provided compelling evidence that including the platform as a covariate did not have a significant influence on prediction accuracy, thereby reinforcing the robustness of the imputation of the integrated dataset (Table S2). This outcome suggests that any platform-specific effects or imputation-related noise were either negligible or did not confound the underlying genetic signals crucial for accurate prediction, affirming the suitability of the integrated dataset for genomic prediction analyses. In addition, we conducted an in-depth comparison of the prediction accuracy for the “Sub” analysis, considering various SNP data scenarios, including the use of the original genotyping SNP data, fully imputed SNPs within each breeding program, and the SNPs in common across all genotyping platforms. These results revealed no significant differences in prediction accuracy when using different SNP sets for any of the evaluated traits (Table S3). This underscores the reliability and consistency of the integrated dataset and affirms its suitability for genomic prediction analyses.

To further demonstrate the benefits of data integration across breeding programs, we conducted another across-breeding program validation strategy that did not require overlap between each program. We began by calculating the genetic correlation between each pair of grain yield trials spanning both programs. Specifically, we identified paired environments across the breeding programs that exhibited a robust genetic correlation above 0.75, and that was at least 0.1 larger than double its estimated standard deviations. We then used the SNP effects derived from one environment to predict the performance of the second environment, which belonged to the other breeding program. This analysis was performed separately using SNP effects calculated within each breeding program, “Sub”, and when using data from both breeding programs in the reference population “All”. This analysis identified nine pairs of environments that met these stringent criteria (Table 3). Noteworthy, the average prediction accuracy across environments was equal to 0.14 when using a reference population from a single breeding program, but increased to 0.20 in the combined analysis that involved data from both breeding programs in the reference population. This highlights the enhanced precision in SNP effect estimation that can be achieved when using a larger reference dataset and emphasizes the potential for more accurate genomic prediction through data collaboration.

**Table 3:** Prediction accuracy of across-breeding program yield trials with high genetic correlation (>0.75) and low standard deviation (two standard deviations at least 0.1 less than the correlation)

Env1-CIMMYT	Env2-USyd	GenCor	Sub	All
BedHeat12	M19.2	0.83 (±0.27)	0.16	0.23
drip13	N15.2	1.00 (±0.31)	0.17	0.22
Flat5IR15	C17.1	0.75 (±0.32)	0.19	0.22
Flat5IR14	N20.1	0.82 (±0.30)	0.22	0.20
Flat5IR12	H19.1	0.86 (±0.36)	0.13	0.20
Jam	N14.2	0.88 (±0.34)	0.11	0.19
Pus	N14.1	0.75 (±0.30)	0.14	0.19
Pus	M19.1	0.93 (±0.27)	0.09	0.16
B2IR16	C17.1	0.78 (±0.27)	0.08	0.15
Average		0.84 (±0.31)	0.14	0.20

*Sub* average prediction accuracy results for each breeding program independently, *All* average prediction accuracy results when analyzing both breeding programs together.

### metaGWAS analysis

**Fig. 2:**
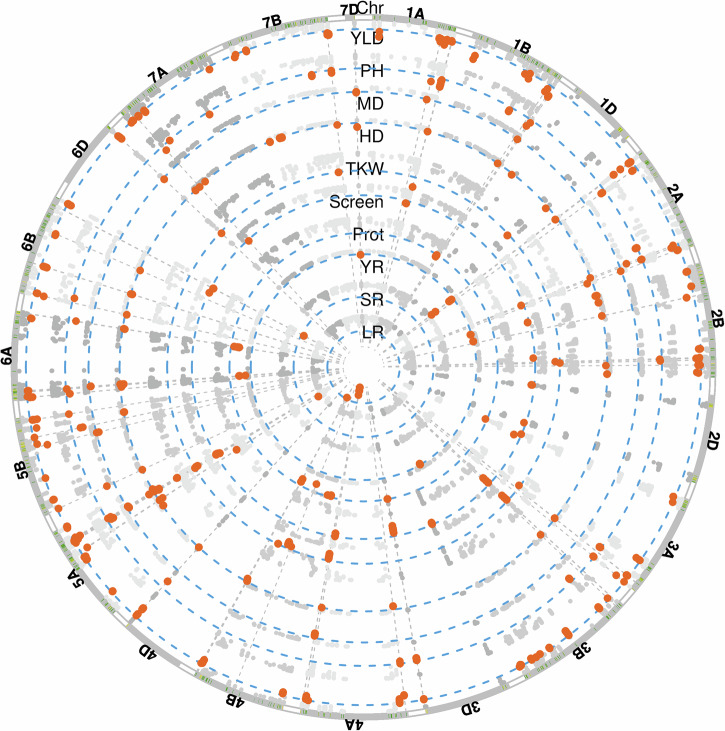
Circular Manhattan plot of the metaGWAS analysis using data from both breeding programs for ten traits. Orange dots represent the significant SNPs. Vertical lines represent multi-environment QTL. HD heading date, MD maturity date, PH plant height, Prot protein content, Screen screening percentage, TKW thousand kernel weight, YLD grain yield, Lr leaf rust, Sr stem rust, Yr yellow rust. Data used to generate the figure can be found in Supplementary data 4.

A metaGWAS analysis conducted across all environments from both breeding programs revealed 118 significant marker-trait associations (Supplementary data 1 and Fig. 2). The number of QTL ranged from two for stem and leaf rust to 75 for grain yield. Among the 118 QTL, 67 were exclusively linked to a single trait while 29, 16, and 4 QTL were associated with two, three, and four traits, respectively. Noteworthy, one QTL located on the long arm of chromosome 1A was associated with five traits, including heading date, plant height, percentage of small seed screening, thousand kernel weight, and grain yield. Another QTL on chromosome 5A (*Vrn-A1*) also exhibited associations with the same five traits and maturity date. In contrast, the same analysis conducted independently within each breeding program resulted in a total of 82 QTL (Supplementary data 2). Remarkably, 78 out of these 82 QTL were also identified in the previous combined analysis, emphasizing the improved statistical power in the combined analysis. Importantly, the 40 additional QTL detected in the joint analysis were primarily associated with grain yield, heading date, and plant height. This underscores the value of integrating data across breeding programs to better understand complex trait associations.

The power analysis revealed that the MetaGWAS framework provided a substantial increase in statistical resolution compared to the single-trait analysis. For example, for grain yield in the present study, the average number of phenotypic records per trial was 922. For such a population, the minimum detectable effect (MDE) required to achieve 80% power at a significance threshold of *α* = 1.5 × 10^−6^ was 3.35% of the total phenotypic variance (Fig. S2). In contrast, the MetaGWAS, which integrated 58,427 phenotypic records, achieved the same power threshold for variants explaining as little as 0.055% of the variance, indicating its ability to detect minor QTL at high power.

## Discussion

Crop breeding programs today are increasingly producing discrete datasets across local environments. Conducting large-scale national or international trials is both costly and logistically demanding, often stretching the limits of available resources within a single program. Therefore, data integration across programs emerges as a solution to these challenges to leverage the genetic and environmental diversity, as well as strengthening cooperation among different breeding programs^20^. Through the integration of diverse data sources, synchronization of genotyping platforms, and the application of efficient computational models like the 3GS method, we have demonstrated its potential to significantly enhance prediction accuracy and unveil valuable trait associations. This study highlights the transformative power of collaborative genomic and statistical efforts in modern plant breeding. We’ve established a robust platform, synchronizing genotype data and developing a novel, efficient computational model by merging data from multiple breeding programs. This collaboration not only enhances prediction accuracy but also uncovers valuable trait associations that can revolutionize breeding strategies. Our findings not only streamline the development of resilient crop varieties but also highlight the critical role of collective data integration in advancing plant breeding for global food security.

One of the primary advantages of data integration lies in the creation of a larger and more genetically and phenotypically diverse reference population to improve selection accuracy and optimize future crosses^21,22^. Moreover, it generates opportunities for the development of climate-resilient cultivars by capturing a wider range of genetic variability and environmental adaptation^23^. Successful data integration for breeding objectives requires two major components. First, synchronization of genotyping platforms across programs if they are different^13^ and second, the development of computationally efficient statistical models to cope with the growing size of data^18^. In this study, we have underscored the pivotal role of data integration in strengthening cooperation between breeding programs through providing a practical example in wheat. The proposed approach offers potential for broad applicability and is extendable to other crop species, animal breeding and human genetics^24^. Moreover, while a diverse reference population is beneficial for capturing broader genetic variability, it is also crucial to ensure sufficient relatedness between the reference and selection candidates for optimal prediction accuracy in elite breeding programs. Our study focuses on pre-breeding, where expanding genetic diversity is often a primary objective. Moreover, the integration of multiple germplasms increases the diversity of the reference population, which should increase the chance of increasing the relatedness with the broader candidate population.

Genetic imputation serves as a valuable tool for synchronizing genotype data across diverse populations^25^. In our study, imputation not only facilitated data integration but also ensured the essential attributes of compatibility and consistency for subsequent analyses. While imputation strategies excel in crops with small genomes where large whole-genome sequenced reference populations are often available to support accurate imputation^26^, their application in wheat currently presents challenges given its large (17GB) genome size^27^. Nevertheless, our results provide compelling evidence that the limited number of SNPs available in this study did not have a significant effect on the prediction accuracy at the individual program level. This can be attributed to the high linkage disequilibrium observed in wheat; a consequence of self-pollination, which usually causes high levels of homozygosity and limited haplotype diversity^28,29^. Thus, large chromosomal segments, sometimes several megabases in size, can be represented by a single SNP^30^, mitigating the impact of differences in marker coverage across platforms after imputation.

The robustness of our imputation process was reinforced by incorporating genotyping platforms as covariates into the genomic prediction models. Our results demonstrate that while GBS imputation accuracy was relatively low for high-MAF variants, this did not negatively impact final genomic prediction results. Including the genotyping platform as a covariate showed no significant influence on accuracy (Table S2), confirming that the imputation process did not introduce confounding biases. The resilience of our prediction models to this localized imputation noise is primarily due to the high LD observed in wheat. In such self-pollinated populations, large chromosomal segments, often several megabases in size, are inherited as blocks. This high LD allows the majority of genetic variance to be captured by a minimal number of accurately genotyped SNPs within each block, effectively mitigating the impact of lower accuracy at specific individual loci within the GBS platform. This finding supports that the observed clustering primarily reflects true genetic differences rather than the confounding effects of the genotyping platforms.

An additional challenge to data integration, particularly when datasets are large, is computational complexity. Standard GxE models often suffer from quadratic increases in computational time as the number of environments increases, unlike the 3GS model, which showed a linear increase^18^. The latter model also allows for high parallelism, making the computational time almost equivalent to analyzing a single environmental trial. The 3GS model offers an efficient solution to the challenges posed by the ever-growing datasets in modern breeding programs, particularly in cases where other models struggle with limited computational resources. Importantly, this advantage extends beyond across-program collaboration; even within a single, large-scale breeding program, the ability to analyze thousands of genotypes across hundreds of historical trials simultaneously without the need for high-performance computing clusters represents a significant shift in operational efficiency.

The original version of the 3GS model^18^ required balanced field trials, as it conducted Principal Component Analysis directly on the phenotypic data. However, achieving such ideal complete phenotypic data is often not realistic in practise, especially when integrating diverse breeding programs^31^. To address this, we adapted the 3GS model to operate on genomic estimated breeding values, which are obtained by initially running a single-environment genomic prediction model for all germplasms, thus ensuring complete datasets. Notably, the key distinction lies in the utilization of the genetic variance covariance matrix instead of the phenotypic variance covariance matrix, as in the original 3GS model. This modification means that the updated 3GS model closely resembles the standard GBLUP model that considers GxE, with the advantage of high computational efficiency. Consequently, it is expected that the prediction accuracy of the 3GS model closely mirrors that of the GxE model across all traits and breeding programs.

The integration of data from both breeding programs yielded significant improvements in prediction accuracy, particularly for traits involving a substantial number of trials across programs. This outcome aligns with previous research demonstrating enhanced prediction accuracy with increased environmental and genotypic diversity^32,33^. To further validate the advantages of data integration, we employed a validation strategy that did not necessitate direct overlap between the breeding programs. By calculating genetic correlations between grain yield trials spanning both programs, even in non-identical environments and using divergent populations, we demonstrated the significant enhancement in prediction accuracy that can be achieved through collaborative data integration. While marker effects can vary across genetically divergent populations, our results demonstrate that leveraging a larger reference population through the 3GS model can still lead to improved across-program prediction accuracy, suggesting that the benefits of increased information outweigh the challenges of population heterogeneity. Such strategies harness broader genetic diversity for prediction equation training, thereby increasing the reference population size in the integrated analysis. This approach facilitates the accurate adaptation of external germplasm across multiple breeding programs.

Data integration significantly improved the statistical power of GWAS analyses^34^, enabling the detection of additional stable QTL across different trials associated with key traits. The increased power in metaGWAS demonstrates that while the smaller single-trial cohort is limited to identifying only major-effect QTL, the vast scale of the integrated dataset enables the detection of the small-effect polygenic QTL, as well as large-effect QTL, that typically characterize the genetic architecture of grain yield. It is worth noting that metaGWAS is powerful to detect small effects and stable QTL across several environments that cannot be detected in small trials, but it fails to detect environmental-specific QTL^35^. Therefore, the choice between the two strategies should depend on the breeding objectives. This heightened statistical power has practical implications for breeding decisions, expediting genetic improvement efforts in wheat breeding programs. However, the high level of LD that was observed in these breeding populations (Fig. S1) limits the resolution for fine mapping. Each QTL could be located within an LD block that reserves tens to hundreds of linked genes. Therefore, QTL identified in this study represent genomic regions rather than specific genes, which is consistent with the primary goal of improving genomic prediction and understanding across-program genetic architecture.

The ability of MetaGWAS to detect QTL explaining as little as 0.055% of the phenotypic variance is a direct result of the expanded sample size (*N* = 58,427). This phenomenon has been extensively documented in human genetics; for example, the captured phenotypic variation for human height increased from 3%^36^ to nearly all SNP-based heritability as the reference population size grew from 13.7 K to 5.4 million individuals^37^. This suggests that much of the missing heritability in complex traits is a function of statistical power and sample size. By integrating datasets across breeding programs, we achieved a resolution that allows for the identification of the small polygenic effects that are typically lost in smaller, isolated studies.

While proprietary concerns might limit data sharing among competing private breeding companies, the benefits of data integration are pronounced for public breeding programs, such as CIMMYT, which often focus on pre-breeding and developing broadly adapted germplasm. For private programs, their own closely related data might indeed be more valuable for predicting elite lines. However, the approach presented here is highly valuable as a pre-breeding strategy, enabling the prediction and incorporation of novel, valuable genetic material into elite breeding programs, thereby broadening the genetic base and accelerating the development of climate-resilient cultivars. This collaborative model offers a pathway to overcome individual program limitations and maximize the impact of breeding efforts.

In summary, the present study highlights the critical importance of data integration in advancing collaboration among inter-institute wheat breeding programs^20^. This collaborative approach not only surmounts logistical and financial challenges but also harnesses broader genetic diversity and environmental variability, offering the prospect of expedited climate-resilient wheat cultivar development and driving progress in genomic prediction and trait association research. Our research serves as a cornerstone for more efficient breeding strategies, ultimately contributing to the creation of superior wheat varieties and boosting global food security. The successful integration of genotypic data from various sources highlights the potential for strengthened collaboration and synergy among breeding programs, emphasizing the importance of cooperative efforts in addressing complex traits crucial for sustainable agriculture and global food security.

## Materials and methods

### Plant materials

The present study used two large populations belonging to two different pre-breeding programs. The first population derived from a breeding program led by the University of Sydney, Australia (USyd), contained a total of 5540 genotypes, of which 2537 lines were genotyped with the wheat 90 K Illumina Infinium array and 3003 doubled haploid lines were genotyped with Illumina wheat-barley 40 K SNP array^38^. Detailed information about the materials, the genotyping and the phenotyping can be found in Joukhadar et al.^35^. The population was phenotyped for heading date (HD), maturity date (MD), plant height (PH), protein content (Prot), screening percentage (Screen), thousand kernel weight (TKW), and grain yield (YLD), as well as the three rust diseases: leaf (Lr), stem (Sr), and yellow rust (Yr). The number of field trials per trait ranged from 6 for the three rusts to 30 for YLD (Table 2), which were scored across different environments in 7 seasons from 2014 to 2020 under normal and late sowing conditions.

The second population was derived from the Global Wheat Program of the International Maize and Wheat Improvement Centre (CIMMYT), including 6069 lines. Detailed information about the materials, the genotyping and the phenotyping can be found in Juliana et al.^39^ and Sehgal et al.^16^. Lines were genotyped via GBS at Kansas State University following the protocol as described in Poland et al.^40^. The population was phenotyped for the same traits as the previous population, except for Lr and Screen. The number of environments per trait ranged from 1 for Prot, TKW and Sr, to 49 for YLD (Table 2), with diverse environmental conditions, including heat, drought and irrigation. To assess the structure and connectivity of the experimental trials, we generated a comprehensive connectivity matrix for all trial pairs within each breeding program (Supplementary data 3). Within both the USyd and CIMMYT programs, trials exhibited high levels of physical overlap and connectivity. For the yield (YLD) trait specifically, the average number of phenotypic records per trial was 442 for USyd and 922 for CIMMYT. Internal trials were well-connected, sharing an average of 136 (30.8%) and 142 (15.4%) common individuals for USyd and CIMMYT, respectively.

Single-nucleotide polymorphism and best linear unbiased estimation (BLUE) for all traits were obtained from the original data holders and used for subsequent analysis^35,39^. The BLUEs were derived from independent replicated single-environment analyses as described in the original publications, and the present study had no access to the original unreplicated datasets. Field trials had at least 98 phenotypic records, with a maximum of 3485 phenotypic records for Yr in Mexico in the CIMMYT program. Across years and field trials within the breeding program, lines generally had high overlap with other trials.

### Imputation

For the process of imputing the three genotyping platforms (90 K and 40 K SNP arrays and GBS) to a higher-density panel, we used a reference population of 868 individuals genotyped with high-density exome capture sequencing SNPs^19^. This involved the following steps^13^:

1. Identification of common SNPs between each of the genotyping platforms and the exome capture sequence data.
2. Selection of SNPs identified by exome capture with a linkage disequilibrium coefficient (*r*^2^) value greater than 0.7 in the reference population, considering the previously identified common SNPs, as the imputation targets.
3. Imputation of missing genotypes in the reference population with exome capture sequencing data using the Beagle5.4 software^41^ with the default settings. Haplotypes were phased using Eagle2.4^42^ with default settings.
4. Imputation to exome SNPs density using Minimac3^43^ and the reference population with default parameters.
5. Selecting only the common high-quality imputed SNPs across the three genotyping platforms for subsequent analyses.

### Imputation accuracy

Estimating imputation accuracy in the present study is challenging, given that there are no common individuals across the germplasms that were genotyped with multiple platforms. Therefore, to estimate the imputation accuracy, we followed the method proposed by Joukhadar et al.^35^. We estimated the concordance between imputed and true genotypes within each germplasm group (genotyped with 90 K, 40 K, or GBS) independently. This was achieved by randomly masking 1% of the overlapping variants between the original platforms and the final imputed set across 1000 replicates. Given these numbers, each variant is expected to be masked on average 10 times across the replicates (1000 × 0.01 = 10). The accuracy was estimated as the average concordance between the imputed call and the true call in this validation strategy. The imputation accuracy of these overlapped variants should provide a rough estimation of the accuracy of imputing the unobserved variants in the final set. To account for the potential bias of high concordance for common alleles, we further partitioned the imputation accuracy results by Minor Allele Frequency (MAF) into different categories (0–0.01, 0.01–0.05, 0.05–0.1, 0.1–0.2, 0.2–0.3, 0.3–0.4, and 0.4–0.5). This allowed us to verify that the imputation process maintained sufficient accuracy even for lower-frequency variants, which are critical for capturing genetic diversity.

### Linkage disequilibrium, SNP-based heritability, and genetic correlation

Linkage disequilibrium (LD) was estimated by calculating the squared correlation coefficient (*r*^2^) for all pairs of intra-chromosomal markers using PLINK v2.0^44^. LD decay was characterized by plotting *r*^2^ values against physical distance for each breeding program and the integrated population. To establish a baseline for background LD, a critical *r*^2^ threshold was determined as the 99th percentile of the *r*^2^ distribution calculated from all unlinked SNP pairs (i.e., markers on different chromosomes).

Variance components were estimated using the GREML model implemented in the MTG2^45^ for each trait in each environment. A genomic relatedness matrix (GRM) was fitted in the model and generated from all SNPs using MTG2 following Yang et al.^46^ as: $G=\frac{Z{Z}^{{\prime} }}{N}$; where **Z** is a matrix of centered marker genotypes; ${Z}^{{\prime} }$ is its transpose, and *N* is the number of markers. These were used to calculate the SNP-based narrow-sense heritability for each trait. The same software was used to run the bivariate REML model to calculate genetic correlations among different environments across both breeding programs.

### Genomic prediction with the GBLUP model

The GE model: The genomic best linear unbiased prediction (GBLUP) was used with the following equation:

$$y=I\mu +E+g+e$$

here, **y** represents the phenotype, respectively, *μ* is the intercept, *g* is the genomic estimated breeding values (GEBVs), and *e* is the random residuals. *E* ~ *N*(0,${\sigma }_{E}^{2}$); *g* ~ (*N*(0,*G*${\sigma }_{g}^{2}$)); and *e* ~ *N*(0,${\sigma }_{e}^{2}$).

The GxE model: The multi-environment GBLUP model described in Hayes et al.^47^ was employed. For two environments, the equation of the model was:

$$\left[\begin{array}{c}{{{{\boldsymbol{y}}}}}_{{{{\bf{1}}}}}\\ {{{{\boldsymbol{y}}}}}_{{{{\bf{2}}}}}\end{array}\right]=\left[\begin{array}{cc}{{{{\boldsymbol{I}}}}}_{{{{\bf{1}}}}} & {{{\bf{0}}}}\\ {{{\bf{0}}}} & {{{{\boldsymbol{I}}}}}_{{{{\bf{2}}}}}\end{array}\right]\left[\begin{array}{c}{{{{\boldsymbol{\mu }}}}}_{{{{\bf{1}}}}}\\ {{{{\boldsymbol{\mu }}}}}_{{{{\bf{2}}}}}\end{array}\right]+\left[\begin{array}{cc}{{{{\boldsymbol{Z}}}}}_{{{{\bf{1}}}}} & {{{\bf{0}}}}\\ {{{\bf{0}}}} & {{{{\boldsymbol{Z}}}}}_{{{{\bf{2}}}}}\end{array}\right]\left[\begin{array}{c}{{{{\boldsymbol{g}}}}}_{{{{\bf{1}}}}}\\ {{{{\boldsymbol{g}}}}}_{{{{\bf{2}}}}}\end{array}\right]+\left[\begin{array}{c}{{{{\boldsymbol{e}}}}}_{{{{\bf{1}}}}}\\ {{{{\boldsymbol{e}}}}}_{{{{\bf{2}}}}}\end{array}\right]$$

where, **y**_**1**_ and **y**_**2**_ represent the phenotype in environment 1 and 2, respectively, **I**_**1**_ and **I**_**2**_ are the identity matrices, μ_1_ and μ_2_ are the intercepts, **Z**_**1**_ and **Z**_**2**_ are the incidence matrices relating phenotypes to genotypes, g_1_ and g_2_ are the GEBVs, and e_1_ and e_2_ are the random residuals.

We assumed that $$\left[\begin{array}{c}{{{{\boldsymbol{g}}}}}_{{{{\boldsymbol{1}}}}}\\ {{{{\boldsymbol{g}}}}}_{{{{\boldsymbol{2}}}}}\end{array}\right]$$~ $${{{\bf{N}}}}\left({{\bf{0, G}}}{{{\boldsymbol{\otimes }}}}\left[\begin{array}{cc}{{{{\boldsymbol{\sigma }}}}}_{{{{\boldsymbol{g}}}}{{{\boldsymbol{1}}}}}^{{{{\boldsymbol{2}}}}} & {{{{\boldsymbol{\sigma }}}}}_{{{{\boldsymbol{g}}}}{{{\boldsymbol{12}}}}}\\ {{{{\boldsymbol{\sigma }}}}}_{{{{\boldsymbol{g}}}}{{{\boldsymbol{12}}}}} & {{{{\boldsymbol{\sigma }}}}}_{{{{\boldsymbol{g}}}}{{{\boldsymbol{2}}}}}^{{{{\boldsymbol{2}}}}}\end{array}\right]\right)$$; **G** is the genomic relatedness matrix, estimated using the method described in VanRaden^48^, ${{{{\boldsymbol{\sigma }}}}}_{{{{\boldsymbol{g}}}}{{{\boldsymbol{1}}}}}^{{{{\boldsymbol{2}}}}}$ and ${{{{\boldsymbol{\sigma }}}}}_{{{{\boldsymbol{g}}}}{{{\boldsymbol{2}}}}}^{{{{\boldsymbol{2}}}}}$ are the variances of environment 1 and 2, ${{{{\boldsymbol{\sigma }}}}}_{{{{\boldsymbol{g}}}}{{{\boldsymbol{12}}}}}$ is the covariance between environment 1 and 2, and ${{{\boldsymbol{\otimes }}}}$ is the Kronecker product. These equations are extendable to any number of environments.

### Genomic prediction with the 3GS model

The 3GS model was previously introduced as a computationally efficient model to run genomic prediction on large multivariate datasets^18^. The original version of the 3GS model lacked the power to analyze unbalanced field trials that do not have full phenotypic records for all individuals in all environments, which limited its applications, given that it is impossible to achieve such data in practical breeding programs. Therefore, we applied the method based on genomic estimated breeding values (GEBVs) calculated for the whole population using SNP effects calculated using a single environment genomic prediction model, which was previously investigated in He et al.^49^ for USyd data and Juliana et al.^39^ for CIMMYT data. The updated 3GS model comprises the following key stages:

1. The Bayesian Ridge Regression (BRR) model, as implemented in the R package Bayesian Generalized Linear Regression, BGLR^50^, was utilized to compute SNP effects independently for each trait in each environment (single-trait analysis) with a total of 20,000 iterations. The initial 10,000 iterations were considered as a burn-in period.
2. Single trait (stGEBV) values for each trait were calculated for all individuals in all environments.
3. Principal component analysis was applied to the stGEBVs of the reference population.
4. SNP effects for each PC were calculated using the BRR model. The analysis involved 20,000 iterations, with the initial 10,000 iterations serving as a burn-in period. By working in the compressed “PC space” rather than the “SNP space,” the 3GS model achieves linear computational complexity, making the integration of massive across-program datasets feasible on standard computing hardware.
5. Calculation of genomic estimated breeding values (pcGEBVs) for the validation population based on the SNP effects for the PCs. This calculation was performed as ${pcGEBV}={{{\boldsymbol{Z}}}}\hat{\beta }$, where *Z* represents the SNP allelic dosage matrix for the validation population, and $\hat{\beta }$ represents the estimated SNP effects from Step 4.
6. Transformation of the pcGEBVs into trait GEBVs for each environment using the equation:

$${{{\rm{GEBV}}}}={{{\boldsymbol{PC}}}}\times {{{{\boldsymbol{R}}}}}^{-{{{\bf{1}}}}}$$

where *PC* is an (*n* × *e*) matrix of pcGEBVs, scaled by multiplying each PC by its respective standard deviation, *n* represents the number of validation individuals, *e* represents the number of environments, and ${{{{\boldsymbol{R}}}}}^{-{{{\bf{1}}}}}$ represents the inverse of the rotation matrix (*e* × *e*), which was scaled by dividing each column by the standard deviation of the corresponding PC. Notably, GEBV is an (*n* × *e*) matrix, resulting in distinct GEBV values for each environment.

Prediction accuracy was assessed by computing the Pearson correlation between GEBV and the actual phenotypic records for each environment. We conducted one hundred random replicates, each time randomly partitioning the population into a reference set (comprising two-thirds of the total population) and a validation set (consisting of the remaining third of the population) before running step 1. Student *t* test was used to declare significance between the prediction accuracies of different prediction models, considering the accuracies of the 100 replicates. Comparisons were declared significant at *p* < 0.01.

### metaGWAS

A single-trait genome-wide association study (GWAS) for each trait within each environment was conducted independently using the following mixed linear equation:

$$y={1}_{n}\mu +X\beta +\alpha +e$$

where, *y* represents a vector of BLUEs (*n* × 1), *μ* is the intercept, *X* is an (*n* × *m*) matrix containing genotyped SNP, *β* is a vector (*m* × 1) denoting SNP allele substitution effects, *α* is a vector (*n* × 1) of random effects, accounting for population structure and relatedness, and e is a vector of random residuals. We assumed that *α* followed a multivariate normal distribution: $\alpha \sim {MVN}(0,\lambda {\tau }^{-1}G)$, where *λ* represents the ratio between two variance components, ${\tau }^{-1}$ denotes the error variance, and *G* is the genomic relatedness matrix calculated as per Yang et al.^46^.

Next, a single *p*-value per SNP was calculated, considering all environments within each breeding program or across both programs using the metaGWAS method described by Bolormaa et al.^51^. This method assumes that the test statistic for multiple traits follows a chi-squared distribution with n degrees of freedom, where *n* corresponds to the number of environments. For each SNP, the chi-squared statistic was calculated using the equation:

$${\chi }_{i}^{2}={t}_{i}^{{\prime} }{V}^{-1}{t}_{i}$$

where, ${t}_{i}$ is a vector of length n, consisting of signed *t*-values for the *i*^th^ SNP across all environments, and ${t}_{i}^{{\prime} }$ represents its transpose. ${V}^{-1}$ represents an (*n* × *n*) matrix, which is the inverse of the correlation matrix among environments. The values of ${t}_{i}$ were computed as:

$${t}_{{ij}}=\frac{{b}_{{ij}}}{{se}({b}_{{ij}})}$$

where ${b}_{{ij}}$ represents the allele substitution effect for SNP *i* in environment *j*, as obtained from the single-trait GWAS analysis, and se(${b}_{{ij}}$) is the standard error of the allele substitution effect. The correlation matrix was calculated as the correlation of *t*-values across different environments. The ${b}_{{ij}}$ values represent the estimated genetic main effects of the SNP within that specific environment. The metaGWAS then combines these environment-specific effects (or their *t*-values) to identify robust associations across multiple environments, effectively capturing main genetic effects that are consistent or show a pattern across environments. SNPs were considered associated and clustered into the same Quantitative Trait Loci (QTL) if they exhibited high linkage disequilibrium with one another (*r*^2^ > 0.5).

### GWAS statistical power analysis

To evaluate the capability of the single-trait GWAS and the metaGWAS analyses to detect genetic associations, we performed a power analysis based on the non-central chi-square (*χ*^2^) distribution. Statistical power, denoted as 1- *β* (where *β* represents the Type II error rate, the probability of failing to detect a true effect), was modeled as a function of the proportion of phenotypic variance (*R*^*2*^) explained by an individual biallelic marker. For a quantitative trait under a linear model, the non-centrality parameter (NCP) was defined as:

$${NCP}=\frac{N{R}^{2}}{1-{R}^{2}}$$

In which, *N* represents the total number of phenotypic records. The power to detect an effect was then calculated as the probability that a random variable from a non-central *χ*^2^ distribution with one degree of freedom exceeds the critical threshold defined by the significance level *α*:

$$\left(1-\beta \right)=P\left({\chi }_{{df}=1,{NCP}}^{2} > {\chi }_{{df}=1}^{2}\right)$$

We set the significance threshold following Bonferroni correction at *α* = 1.5 × 10^−6^ (0.05/32,822). The Minimum Detectable Effect (MDE) was defined as the minimum *R*^*2*^ required to achieve a statistical power of 0.8. All power calculations and visualizations were performed using the scipy.stats library in Python 3.

### Statistics and reproducibility

All statistical analyses (except for the 3GS model developed here) were conducted using established genomic software and custom R scripts. Variance components and SNP-based narrow-sense heritability were estimated using the GREML model in MTG2^45^. Genetic correlations between environments were calculated using bivariate REML in the same software, and both analyses were conducted with the default parameters. For genomic prediction, we employed the standard GBLUP models (GE and GxE) and the updated 3GS model implemented through the BGLR statistical R pacakge. The 3GS model utilized Bayesian Ridge Regression (BRR) via the BGLR R package, with 20,000 iterations and a 10,000 iteration burn-in period to ensure parameter convergence and default values for all other parameters^50^. Linkage disequilibrium (LD) was estimated using PLINK v2.0^44^.

The study utilized a total sample size of 11,609 wheat accessions, 5540 from the University of Sydney (USyd) and 6069 from CIMMYT, which were previously published^39,49^. Phenotypic data were integrated from 79 environments across both sets. For USyd, trials consisted of 6 to 30 environments per trait across seven seasons (2014–2020), while CIMMYT trials included 1 to 49 environments per trait. The number of phenotypic records per trial ranged from 98 to 3485.

Reproducibility was ensured through standardized data processing and cross-validation strategies:

- Phenotypic data: Analyses were based on Best Linear Unbiased Estimates (BLUEs) derived from independent, replicated single-environment trials as described in original source publications.
- Imputation accuracy: To validate the genotype imputation, we performed 1000 random replicates, masking 1% of overlapping variants to calculate concordance between imputed and true genotypes.
- Model validation: Genomic prediction accuracy was assessed using 100 random replicates for each trait and model, where the population was partitioned into reference and validation sets to ensure the robustness of the findings.

## Supplementary information

Supplementary Information

Description of Additional Supplementary Files

Supplementary Data 1–3

Supplementary Data 4

reporting-summary

## Data Availability

Processed data as well as raw data from the University of Sydney are available at: Jighly et al.^38^ (10.5281/zenodo.19217089). CIMMYT data were previously published in Juliana et al.^39^ (Associated data with the paper available at: 10.6084/m9.figshare.8940257.v1).

## Code availability

Codes are available at: Jighly et al.^38^ (10.5281/zenodo.19217089) as well as: https://github.com/ajighly/3GS.
