# Supplementary material for: Large scale wheat data integration improves genomic prediction accuracy with the potential to facilitate international breeding collaborations
Source: Commun Biol. 2026 Apr 28;9:892. doi: 10.1038/s42003-026-10150-x (PMC13332232; doi:10.1038/s42003-026-10150-x)
Supplement: Supplementary file 1 — Supplementary Information [file 42003_2026_10150_MOESM1_ESM.pdf]

Supplementary materials

Figure S1. Linkage disequilibrium (LD) decay for the materials genotyped with each genotyping platform (90K, 40K, and GBS) as well as the combined LD decay with all of them together.

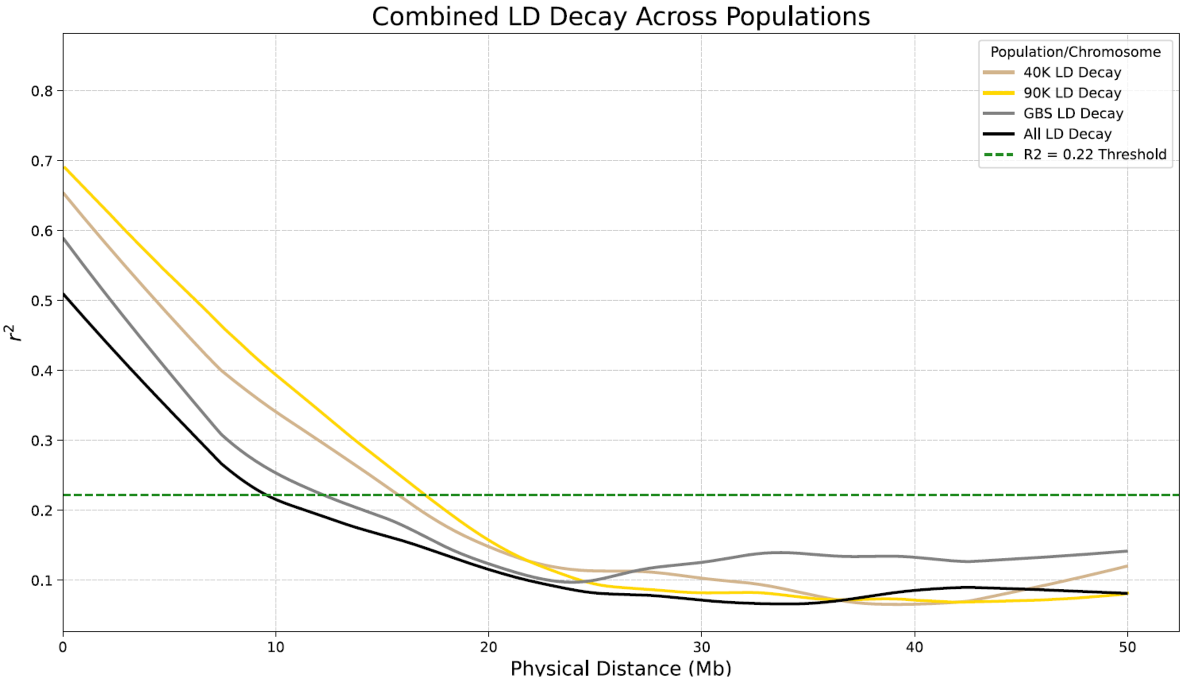

Figure S2. GWAS power analysis for grain yield trait (GY) considering the metaGWAS analysis that integrates all field trials across both breeding programs and the average population size of all single-environment trials (922 individuals).

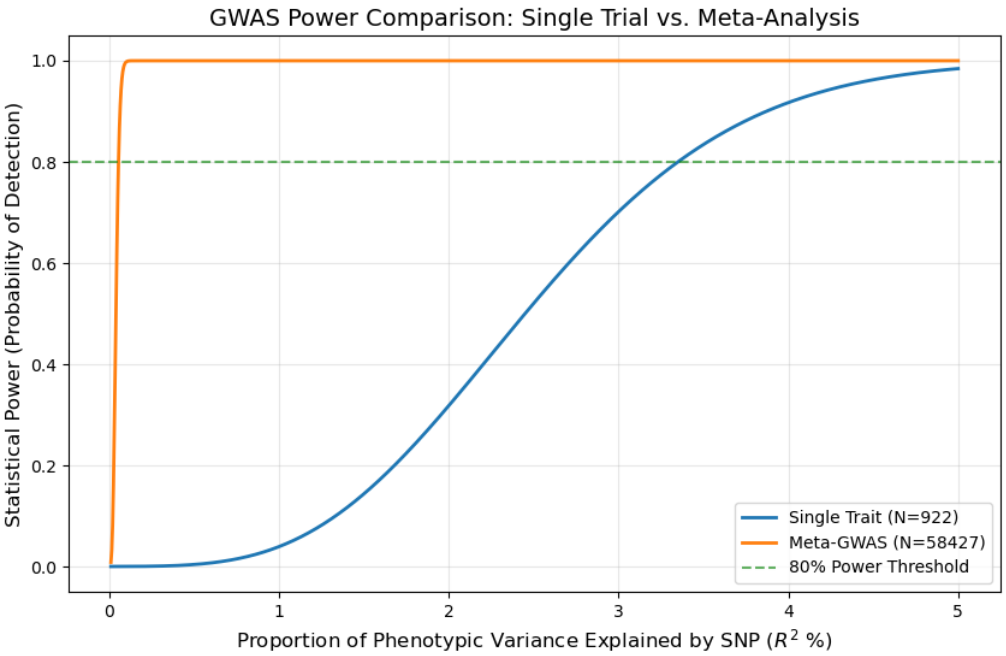

Table S1. Imputation results against minor allele frequency (MAF) of the imputed SNPs across the three genotyping platforms (90K, 40K, and GBS)

MAF Range	OverAll	90K	40K	GBS
0.00-0.01	0.96	0.97	0.97	0.95
0.01-0.05	0.94	0.98	0.96	0.92
0.05-0.10	0.91	0.99	0.93	0.87
0.10-0.20	0.87	0.96	0.90	0.81
0.20-0.30	0.81	0.95	0.86	0.71
0.30-0.40	0.76	0.95	0.85	0.62
0.40-0.50	0.73	0.95	0.84	0.56

Table S2. Average genomic prediction accuracy across different environments for ten commercial traits using three different models: GE, GxE and 3GS when using the genotyping platform (90K, 40K or GBS) as fixed covariate.

Trait	GE		GxE		3GS	
	Sub	All	Sub	All	Sub	All
**HD**	0.27	0.36	0.37	NA	0.42	0.46
**MD**	0.45	0.49	0.49	0.53	0.48	0.53
**PH**	0.40	0.39	0.44	NA	0.42	0.47
**Prot**	0.44	0.46	0.56	0.53	0.53	0.56
**Screen**	0.31	NA	0.44	NA	0.46	NA
**TKW**	0.58	0.62	0.65	0.66	0.64	0.65
**YLD**	0.19	0.23	0.35	NA	0.39	0.39
**Lr**	0.43	NA	0.50	NA	0.47	NA
**Sr**	0.35	0.39	0.42	0.48	0.46	0.49
**Yr**	0.40	0.46	0.46	0.50	0.45	0.50

Sub: average prediction accuracy results for each breeding program independently; All: average prediction accuracy results when analysing both breeding programs; NA: analysis was not possible due to the lack of records in one dataset or due to computational limitation; HD: heading date; MD: maturity date; PH: plant height; Prot: protein content; Screen: screening percentage; TKW: thousand kernel weight; YLD: grain yield; Lr: leaf rust; Sr: stem rust; Yr: yellow rust.

Table S3. Average genomic prediction accuracy across different environments for ten commercial traits using three different models: GE, GxE and 3GS, when using each breeding program independently with three sets of SNPs. 1) Original genotyping, 2) full imputed genotyping to exome level, and 3) the overlapped set of SNPs that was used for the combined analysis

Trait	GE			GxE			3GS		
	Original	Imputed	Overlapped	Original	Imputed	Overlapped	Original	Imputed	Overlapped
**HD**	0.29	0.32	0.29	0.40	0.40	0.39	0.40	0.41	0.41
**MD**	0.45	0.44	0.44	0.49	0.48	0.47	0.50	0.49	0.48
**PH**	0.39	0.41	0.39	0.46	0.45	0.44	0.45	0.46	0.44
**Prot**	0.45	0.48	0.45	0.55	0.56	0.54	0.56	0.56	0.53
**Screen**	0.33	0.35	0.32	0.45	0.48	0.45	0.44	0.48	0.47
**TKW**	0.59	0.63	0.6	0.63	0.64	0.64	0.62	0.64	0.65
**YLD**	0.23	0.24	0.21	0.37	0.40	0.37	0.37	0.41	0.37
**Lr**	0.46	0.45	0.45	0.48	0.50	0.48	0.47	0.51	0.48
**Sr**	0.36	0.38	0.35	0.44	0.44	0.43	0.44	0.44	0.44
**Yr**	0.42	0.42	0.41	0.46	0.47	0.47	0.45	0.47	0.47

HD: heading date; MD: maturity date; PH: plant height; Prot: protein content; Screen: screening percentage; TKW: thousand kernel weight; YLD: grain yield; Lr: leaf rust; Sr: stem rust; Yr: yellow rust.
