# Supplementary material for: Large scale wheat data integration improves genomic prediction accuracy with the potential to facilitate international breeding collaborations
Source: Commun Biol. 2026 Apr 28;9:892. doi: 10.1038/s42003-026-10150-x (PMC13332232; doi:10.1038/s42003-026-10150-x)
Supplement: Supplementary file 2 — Description of Additional Supplementary Files [file 42003_2026_10150_MOESM2_ESM.pdf]

Description of Additional Supplementary files

File name: Supplementary Data 1

Description: Significant marker trait associations detected with metaGWAS analysis that considers data from both pre-breeding programs

File name: Supplementary Data 2

Description: Significant marker trait associations detected with metaGWAS analysis that considers data from each pre-breeding programs independently

File name: Supplementary Data 3

Description: Number of phenotypic records and number of overlapped individuals across different traits/trials

File name: Supplementary Data 4

Description: Source data for figure 2.
